# Influence of light-curing mode on the cytotoxicity of resin-based surface sealants

**DOI:** 10.1186/1472-6831-14-48

**Published:** 2014-05-06

**Authors:** Florian J Wegehaupt, Tobias T Tauböck, Thomas Attin, Georgios N Belibasakis

**Affiliations:** 1 Clinic for Preventive Dentistry, Periodontology and Cariology, University of Zurich, Plattenstrasse 11, 8032 Zürich, Switzerland; 2 Section of Oral Microbiology and Immunology, Institute of Oral Biology, University of Zürich, Plattenstrasse 11, 8032 Zürich, Switzerland

**Keywords:** Dentine, Surface sealant, Light exposure time, Resin, Cytotoxicity

## Abstract

**Background:**

Surface sealants have been successfully used in the prevention of erosive tooth wear. However, when multiple tooth surfaces should be sealed, the light-curing procedure is very time-consuming. Therefore, the aim of this study was to investigate whether reduced light-curing time (while maintaining similar energy density) has an influence on resin-based surface sealant cytotoxicity.

**Methods:**

Bovine dentine discs were treated as follows: group 1: untreated, groups 2–5: Seal&Protect and groups 6–9: experimental sealer. Groups 2 and 6 were light-cured (VALO LED light-curing device) for 40 s (1000 mW/cm^2^), groups 3 and 7 for 10 s (1000 mW/cm^2^), groups 4 and 8 for 7 s (1400 mW/cm^2^) and groups 5 and 9 for 3 s (3200 mW/cm^2^). Later, materials were extracted in culture medium for 24 h, and released lactate dehydrogenase (LDH) activity as a measure of cytotoxicity was determined photometrically after cells (dental pulp cells and gingival fibroblasts) were exposed to the extracts for 24 h. Three independent experiments, for both sample preparation and cytotoxicity testing, were performed.

**Results:**

Overall, lowest cytotoxicity was observed for the unsealed control group. No significant influence of light-curing settings on the cytotoxicity was observed (p = 0.537 and 0.838 for pulp cells and gingival fibroblasts, respectively). No significant difference in the cytotoxicity of the two sealants was observed after light-curing with same light-curing settings (group 2 vs. 6, 3 vs. 7, 4 vs. 8 and 5 vs. 9: p > 0.05, respectively).

**Conclusions:**

Shortening the light-curing time, while maintaining constant energy density, resulted in no higher cytotoxicity of the investigated sealants.

## Background

Erosive dental hard tissue loss or softening can be prevented by topical application of various chemical compounds (e.g. amine fluoride [1] and sodium fluoride [2]). This softening or loss of dental hard tissues could also be prevented by hampering the contact of the erosion causing acids with the dental hard tissues by means of a mechanical barrier. Brunton et al. [3] suggested a coating of erosive exposed dentine with a resin-based dentine adhesive to prevent further wear. Recent studies have shown a good protective effect of such a coating against erosive and erosive/abrasive wear under both *in-vivo* and *in-vitro* conditions [4-7].

However, the sealant (Seal&Protect, DENTSPLY DeTrey, Konstanz, Germany) used in these studies has to be light-cured for at least two times for 10 s per tooth surface, when using common polymerisation device settings (approximately 1000 mW/cm^2^ output intensity). When multiple tooth surfaces have to be sealed, the light-curing procedure becomes very time-consuming. In a recent study, surface sealants have been light-cured with a shorter duration, while the light intensity was simultaneously increased [8]. In that study, no significant difference in the protective effect against erosive demineralization and in the mechanical stability was observed, when the surface sealants were fast light-cured.

For other resin-based dental materials (adhesives, composites and luting cements) a shorter light-curing duration usually results in a lower degree of conversion [9-11], inferior mechanical properties [12] and a higher cytotoxicity [13,14]. With the development of increased intensity light-curing units, one might consider compensating for lower degrees of conversion due to shorter light-curing durations by increasing the light-curing intensity. However, there are studies [15,16] showing that a massive increase in the light-curing intensity may have an adverse effect on the degree of conversion, with higher light intensity resulting in a lower degree of conversion. Beside poorer mechanical properties [12,17], a lower degree of conversion (higher amount of remaining unpolymerised monomers) is also associated with a higher cytotoxicity of resin-based materials [18-20].

To the present, no study has been published that examines the use of increased light intensity to compensate for the negative side effects (increased cytotoxicity) of a shortened light-curing duration of surface sealants. If this shortening is possible without negative effects (this means increased cytotoxicity), this might reduce the time consumed in the procedure, when sealing is applied in whole dentition or numerous teeth.

Therefore, the aim of the present study was to investigate whether shortening the light-curing duration (while maintaining similar energy density by simultaneously increasing the light-curing intensity) would have an influence on the cytotoxicity of two tested surface sealants.

The hypothesis of the present study was that (1) shortening the light-curing time while simultaneously increasing light intensity results in a higher cytotoxicity of the surface sealants and (2) that the type of sealer has an influence on cytotoxicity.

## Methods

### Sample preparation

For this study, 126 bovine dentine discs were prepared from bovine lower incisors roots. The bovine teeth were collected as anonymous by-products of regular slaughtering of the cattle. Slaughtering was performed to provide the cattle as foodstuff for human consumption. Therefore, no ethic approval was needed. Samples were extracted with a trephine drill (inner diameter of the drill: 5 mm) from the distal and mesial surface of each root. The drilling cores were ground down to a thickness of 1 mm with abrasive paper (800, 1000, 1200, 2400, and 4000 grit; Water Proof Silicon Carbide Paper, Streuers, Erkrat, Germany). After grinding, the samples were checked with a stereomicroscope at 40× magnification to ensure complete cementum removal. After preparation, the dentine discs were gamma sterilized (12 kGy, 4 h, Paul Scherrer Institut, Villigen, Switzerland) while stored in water and further on stored in tap water until they were used in the study. The 126 dentine discs were randomly allocated to nine groups (1 – 9, n = 14).

### Sealing procedure

The surface sealants, their composition, their batch numbers and the manufacturers are given in Table 1.

**Table 1:** Composition of the used surface sealants (manufacturer’s information)

**Product**	**Composition**
Seal&Protect (DENTSPLY DeTrey, Konstanz, Germany) Lot: 1203000305	Di- and trimethacrylate resins, PENTA (dipentaerythritol penta acrylate monophosphate), functionalised amorphous silica, photoinitiators, butylated hydroxytoluene, cetylamine hydrofluoride, triclosan, acetone
K-0184 (DENTSPLY DeTrey, Konstanz, Germany) Lot: LAN-18-153-1	Di- and trimethacrylate resins; PENTA (dipentaerythritol penta acrylate monophosphate), functionalised amorphous silica, photoinitiators, butylated hydroxytoluene, cetylamine hydrofluoride, acetone

The dentine discs of group 1 were left untreated and served as untreated control. The dentine discs of groups 2 – 5 were treated with Seal&Protect (DENTSPLY DeTrey, Konstanz, Germany), whereas the dentine discs in groups 6 – 9 were treated with K-0184 (experimental sealer; DENTSPLY DeTrey). The respective sealants (one drop per application) were applied on the surface of the dentine disc and left undisturbed for 20 s. After 20 s, the remaining solvent was removed with an air syringe, and the sealant was light-cured. A second layer of sealant was applied, the solvent evaporated with an air syringe and light-cured again. Light-curing was performed with the VALO LED light-curing device (Ultradent Products, South Jordan, USA). In groups 2 and 6 light-curing was performed at standard mode (1000 mW/cm^2^) for 40 s (=40 J/cm^2^), in groups 3 and 7 at standard mode (1000 mW/cm^2^) for 10 s (=10 J/cm^2^), in groups 4 and 8 at high power mode (1400 mW/cm^2^) for 7 s (=9.8 J/cm^2^) and in groups 5 and 9 at plasma-emulation mode (3200 mW/cm^2^) for 3 s (=9.6 J/cm^2^). The light-curing unit was checked for consistency prior to curing using a radiometer (Optilux Radiometer, SDS Kerr; Orange, CA, USA). Holding the samples with a forceps and resting the light output window on the forceps guaranteed a constant distance between light-curing tip and samples surface of 0.5 cm [8]. After the final polymerisation, the oxygen-inhibited (soft surface) layer was removed with a cotton pellet.

### Preparation of extracts

The 14 samples per group were transferred into one well (22.1 mm diameter) of a 12 well cell culture plate (SPL Life Sciences Inc., Gyeonggi-do, South Korea). During transferring the dentine discs in the wells, care was taken that the discs were placed with the sealed side up in the well. The dentine discs were covered with 3 ml cell culture medium, consisting of DMEM/F12 medium, supplemented with 1% penicillin/streptomycin, 1% L-glutamine, 50 ng/ml fungizone and 10% heat-inactivated foetal bovine serum (all from Sigma-Aldrich, Buchs, Switzerland). They were thereafter incubated in the dark for 24 h at 37°C and 5% CO_2_[21]. Thus, extracts of the dentine discs were prepared at a ratio of 91.6 mm^2^ sample surface per millilitre cell culture medium following the recommendations of ISO [21].

### Cell cultures

Human dental pulp cells from permanent teeth and gingival fibroblasts were obtained according to previously described procedures and ethical requirements [22,23]. The gingival fibroblasts were provided by Dr. Anders Johansson, Institute of Odontology, Umeå University, Sweden (Human Studies Ethical Committee of Umeå University, Sweden - §68/03, dnr 03–029). The collection of dental pulp cells abides by guidelines of the Ethical Committee of the Canton of Zürich, Switzerland, for collection of material for research purposes obtained from discarded and irreversibly anonymized specimens of human origin. For the experimentations in the present study, the cells were cultured in DMEM/F12 medium, supplemented with 1% penicillin/streptomycin, 1% L-glutamine, 50 ng/ml fungizone and 10% heat-inactivated foetal bovine serum (all from Sigma-Aldrich, Buchs, Switzerland). For the experimentations, cell cultures between the third and fifth passages were used. Dental pulp cells were seeded at a density of 2 × 10^5^ cells per well, whereas gingival fibroblasts were seeded at a density of 1.2 × 10^5^ cells per well (four replicate cultures per extract dilution group) of a 96-well plate, and incubated for 24 h at 37°C to allow for cell attachment on the bottom of the well, reaching 100% confluence. Thereafter, 200 μl per well of the extracts were added to the cell cultures, and allowed to incubate for 24 h, in order to investigate cytotoxicity.

### Cytotoxicity assay

The potential cytotoxic effects of different treatment groups on dental pulp cell and gingival fibroblast cultures were evaluated by measurement of the extracellularly released cytosolic lactate dehydrogenase (LDH), using the CytoTox96^®^ Non-Radioactive Cytotoxicity Assay (Promega Dübendorf, Switzerland). After the 24 h exposure of the cell cultures to the material extracts, the cell culture supernatants were collected, while the adherent cells were lysed by three repeated cycles of freeze-thawing, in 200 μl of cell culture media. Both the cell supernatants and lysates were centrifuged at 1000 rpm for 5 min to remove any cell debris, and thereafter diluted 1:10 and transferred into an optically clear 96-well plate, followed by addition of reaction solution and incubated for 30 min in the dark. The reaction was then stopped and the absorbance was measured at 490 nm in an Epoch microplate reader (Biotek, Lucerne, Switzerland), subtracting the corresponding background values from all samples. The cytotoxicity results are expressed as percentage of extracellularly released LDH activity, calculated against total (intracellular + extracellular) LDH activity. This percentage corresponds to the relative amount of dead cells among the total cells in culture. Three independent experiments were performed (including both sample preparation and cytotoxicity testing).

### Statistical analysis

For each of the three independent experiments, the mean percentage of released LDH of the four biological replicates per group was calculated and later used as values for the respective group in respective experiment. For statistical analysis the mean percentage of the respective values (mean of the four biological replicates per experiment) of the three experiments was calculated.

The statistical analysis was performed using the software program IBM^®^ SPSS^®^ Statistics Version 22 (Internetional Business Machines Corp., Armonk, New York, United States).

The assumption of normal distribution of errors was checked, using Shapiro-Wilk test.

Statistical analysis was performed by 2-way ANOVA with the factors light-curing setting and sealer separately for dental pulp cells and gingival fibroblasts followed by Bonferroni/Dunn post-hoc test. Level of significance was set at p < 0.05.

## Results

### Extracellularly released LDH activity from dental pulp cells

**Figure 1:**
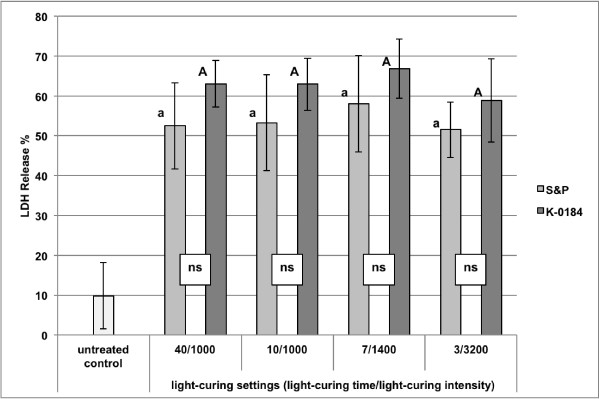
**LDH release from dental pulp cells.** Detailed legend: Percentage (mean ± SD) of extracellularly released LDH activity from dental pulp cells for different sealants (S&P = Seal&Protect and experimental sealant K-0184) and different light-curing settings (light-curing duration in s/light-curing intensity in mW/cm^2^). Comparisons within the same sealant between the different light-curing settings that are not significantly different, are marked with same letters (lower case letters and capital letters for S&P and K-0184, respectively). Comparisons within the same light-curing setting between the different sealants that are not significantly different, are marked with ns.

The percentage of extracellularly released LDH from pulp cells for the different groups treated with different sealants and different light-curing settings are presented in Figure 1.

No significant influence of the light-curing setting (p = 0.537) on the cytotoxicity of the surface sealants was observed. The type of sealant, however, had a significant influence on the cytotoxicity could be observed (p = 0.018).

The significantly lowest cytotoxicity was observed for the untreated control group 1.

The 2-way ANOVA showed a significant influence of the type of sealant on the cytotoxicity. Within the respective light-curing settings, K-0184 showed a higher cytotoxicity than Seal&Protect, however these differences were not statistically significant (group 2 vs. 6: p = 1.000; 3 vs. 7: p = 1.000; 4 vs. 8: p = 1.000 and 5 vs. 9: p = 1.000).

### Extracellularly released LDH activity from gingival fibroblasts

**Figure 2:**
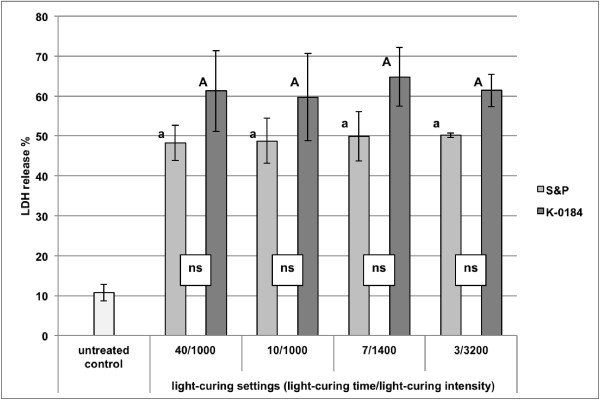
**LDH release from gingival fibroblast.** Detailed legend: Percentage (mean ± SD) of extracellularly released LDH activity from gingival fibroblast for different sealants (S&P = Seal&Protect and experimental sealant K-0184) and different light-curing settings (light-curing duration in s/light-curing intensity in mW/cm^2^). Comparisons within the same sealant between the different light-curing settings that are not significantly different, are marked with same letters (lower case letters and capital letters for S&P and K-0184, respectively). Comparisons within the same light-curing setting between the different sealants that are not significantly different, are marked with ns.

The percentage of extracellularly released LDH from gingival fibroblasts for the different groups treated with different sealants and different light-curing settings are presented in Figure 2.

No significant influence of the light-curing setting (p = 0.838) on the cytotoxicity of the surface sealants was observed. When comparing the types of sealant, a significant influence on the cytotoxicity was observed (p < 0.001).

The significantly lowest cytotoxicity was observed for the untreated control group 1.

Although 2-way ANOVA showed a significant influence of the type of sealant on the cytotoxicity, no significant difference could be observed between the two sealants light-cured with the same light-curing setting (group 2 vs. 6: p = 0.995; 3 vs. 7: p = 1.000; 4 vs. 8: p = 0.507 and 5 vs. 9: p = 1.000). However, K-0184 showed a higher tendency towards cytotoxicity than did Seal&Protect within the same light-curing setting.

## Discussion

In the present study, the extracts of the respective sealants were prepared by immersing dentine discs covered with the different sealants in cell culture medium. Other studies investigating the cytotoxicity of dental materials e.g. adhesives prepared the extracts by either curing the materials to be tested in vials [24,25], wells [26], on glass slides [27] or in moulds [28]. Later the cell culture medium was exposed to these cured materials or the uncured materials were directly given to the cells [29] or were dissolved in cell culture medium [30]. The disadvantage of curing the materials in vials or wells (e.g. well of 96-well microplates) is that the oxygen inhibition layer, rich on uncured monomers and adhesive components, on top of these materials cannot be easily removed. As the uncured monomers and other components can be easily diluted from the oxygen inhibition layer, this results in an increased amount of these substances in the extracts prepared from samples in which the oxygen inhibition layer is not removed. There are also disadvantages in curing the materials on glass slides or in moulds. These materials, as well as the sealants tested here, are purported to react with the dental hard substances they are applied on. During this reaction it might be assumed that there is a change in the chemical composition or reactiveness of the applied materials. This change might have an influence on the later release of possible cytotoxic compounds from the materials. Therefore, we assume that applying the sealants on dentine discs and removing the oxygen inhibition layer, as recommended by the manufacturer, before immersing them in cell culture medium is advantageous for the health of the tissue.

The dentine used for preparation of the dentine discs was gained from bovine teeth. Although reaction with and the effect upon human dentine is the actual target of dental research, there are numerous advantages of using bovine dentine. On the one hand it is easy to obtain a sufficient number of sound bovine teeth [31] and due to their larger surface, multiple samples can be gained from one tooth resulting in a lower baseline diversity of the samples. On the other hand, bovine teeth do not have a fluoride and/or caries history that many human teeth have, which might influence their chemical composition and the interaction with applied surface sealants.

To test the cytotoxic effect of the surface sealants, dental pulp cells and gingiva fibroblasts were used. We assume that under clinical conditions these cell types are the ones mainly affected by the cytotoxicity of surface sealants. Furthermore, dental pulp cells [21,23,32,33] and gingival fibroblasts [18,22,26,34] have been used in numerous other studies testing the cytotoxicity of resin-based dental materials, or various other biological agents. Measurement of the activity of extracellular released LDH is a very suitable routine assay for evaluating the cytotoxic effects of various agents, including chemicals or bacteria, on eukaryotic cells [35,36]. Nevertheless, it should be noted that the assay can measure death by cell lysis, rather than the more perplexed mechanistically apoptotic cell death, which would be beyond the scope of the present study.

One limitation of the present study might be that during application of the sealants no intra-pulpal pressure was simulated. The resulting outwards directed flow of dentine liquor might decrease the contact of pulp cells with the sealants applied on the dentine. Taking into consideration these findings, we assume that the values gained for the cytotoxicity on dental pulp cells might be slightly over-estimated. However, if the sealants would have been applied in a dentine barrier test setup like that used in other studies testing the cytotoxicity of adhesive systems [37,38] there might well have been an under-estimation of the cytotoxicity on gingival fibroblasts. Therefore, we considered an over-estimation of the cytotoxicity on dental pulp cells to be more acceptable than an under-estimation of the cytotoxic effect on gingival fibroblasts.

The primary hypothesis of the present study, that shortening the light-curing time while simultaneously increasing the light intensity results in a higher cytotoxicity of the surface sealants, has to be rejected as no significant influence of the light-curing settings on the cytotoxicity was observed, neither for the dental pulp cells nor for the gingival fibroblasts. This finding is in contrast with other studies showing that for other resin-based dental materials (composites and luting cements) a shorter light-curing duration most likely results in a higher cytotoxicity [13,14]. We assume that there are two possible explanations for these contrary findings: On one hand, the light-curing time was shortened, but simultaneously, the light-curing intensity was increased, resulting in an application of similar energy densities, while in the above mentioned studies only the light curing time was shortened. It can be assumed that the application of similar energy densities results in the formation of similar amounts of radicals, resulting in a similar degree of conversion. On the other hand, in those studies that showed a higher cytotoxicity after shorter light-curing time, restorative resin composites [14] or resin luting cements [13] with a thickness of the samples of 2 mm and 1 mm were tested, respectively. In contrast, the layer thickness of the sealants tested in the present study commonly amounted to between 22 μm [39] to 40 μm [40]. We assume that due to this much lower thickness of the sealant’s layer, shortening of the light-curing duration has a lower influence on the cytotoxicity, as the curing light can easily reach the bottom side of the samples (sealant layer). When the curing light easily reaches even the bottom side of samples shorter light-curing duration will deliver enough energy to these areas of the material to ensure an adequate curing of the monomers. All surface sealants groups (irrespective of the light-curing settings used) showed a significant cytotoxicity. However, in none of the “fast” light-curing groups (4, 5, 8 and 9) was a significantly higher cytotoxicity observed than for the groups light-cured following the manufacturers’ instructions (groups 3 and 7) or the groups with extensive light-curing (groups 2 and 6). To the best of our knowledge, there have been no reported issues with biocompatibility of sealants tested when light-curing following the manufacturers’ instructions, so that one might assume that the human organism can tolerate the here found cytotoxicity.

Also the secondary hypothesis that the type of sealer has an influence on the cytotoxicity, has to be rejected. Although the ANOVA showed a significant influence of the kind of sealant on the cytotoxicity for both dental pulp cells and gingival fibroblasts, no significant difference in the cytotoxicity of the sealants could be observed when comparing the values of the two sealants irradiated with identical curing protocols. However, a higher, but not distinctly different, cytotoxicity could be observed for K-0184 for both cell types. This finding might be attributed to the composition of the material. Basically, K-0184 has the same formulation as Seal&Protect, but contains no triclosan. Triclosan is incorporated as an antimicrobial additive in numerous personal care and sanitizing products such as soaps, household cleaners, cosmetics, sportswear, mouthwash and toothpaste [41]. Concerns about the use of triclosan have been raised as studies have shown that triclosan is able to induce antibiotic resistances in various bacteria stems [42], to accumulate in human milk samples and in fish exposed to municipal wastewater [43]. The easiest way to prevent these negative side effects of triclosan is to avoid incorporating it into products used in human subjects. However, if the triclosan is excluded from the given chemical formulation of Seal&Protect, this will also change the proportion of the other components in the formulation. It might be assumed, that this change might result in a shift or increase of unreacted components in the light-cured product, subsequently causing a higher cytotoxicity.

## Conclusion

Within the limitations of the present study it can be concluded that shortening the light exposure time, while maintaining the energy density, resulted in no higher cytotoxicity of the surface sealants cured in this manner. Taking further in consideration that shortening the light exposure times, while maintaining the energy density, has no negative influence on the erosion prevention potential and mechanical stability of the surface sealants [8] it may be concluded that the light-curing protocol (3 s light-curing at 3200 mW/cm^2^ (=9.6 J/cm^2^)) used here provides a fast and safe approach to use surface sealants to prevent erosive tooth wear. However, further studies regarding abrasion, degree of conversion and long-term stability of so cured surface sealants are needed before recommendations for clinical use can be given.

## Competing interests

The authors declare that they have no competing interests.

## Authors’ contributions

FJW participated in the study design of the study, preparation of the tested material, performance of cell cultures, data analysis and wrote the manuscript. TTT helped in writing the manuscript and gave especially critical input concerning the polymerisation topic. TA participated in the design of the study and critically reviewed the article. GNB participated in the study design, performance of the cell cultures and LDH assays, data analysis and critically reviewed the manuscript. All authors read and approved the final manuscript.

## Pre-publication history

The pre-publication history for this paper can be accessed here:

http://www.biomedcentral.com/1472-6831/14/48/prepub
